# Genetic counseling of patients with ovarian carcinoma: acceptance, timing, and psychological wellbeing

**DOI:** 10.1007/s12687-019-00427-6

**Published:** 2019-06-05

**Authors:** I. Van de Beek, E. M. A. Smets, M. A. Legdeur, J. A. de Hullu, C. A. R. Lok, M. R. Buist, M. J. E. Mourits, C. M. Kets, L. E. van der Kolk, J. C. Oosterwijk, C. M. Aalfs

**Affiliations:** 1 Department of Clinical Genetics, Amsterdam UMC, location VUmc, PO Box 7057, 1007 MB Amsterdam, The Netherlands; 2 Department of Clinical Genetics, Amsterdam UMC, location AMC, PO Box 22660, 1100 DD Amsterdam, The Netherlands; 3 Department of Medical Psychology, Amsterdam UMC, location AMC, PO Box 22660, 1100 DD Amsterdam, The Netherlands; 4 Department of Obstetrics and Gynaecology, Radboud University Medical Center, PO Box 9101, 6500 HB Nijmegen, The Netherlands; 5 Department of Obstetrics and Gynaecology, Center of Gynaecologic Oncology Amsterdam, PO Box 90203, 1006 BE Amsterdam, The Netherlands; 6 Department of Obstetrics and Gynaecology, Academic Medical Center, PO Box 22660, 1100 DD Amsterdam, The Netherlands; 7 Department of Obstetrics and Gynaecology, University Medical Center Groningen, University of Groningen, PO Box 30001, 9700 RB Groningen, The Netherlands; 8 Department of Human Genetics, Radboud University Medical Center, PO Box 9101, 6500 HB Nijmegen, The Netherlands; 9 Family Cancer Clinic, Netherlands Cancer Institute, PO Box 90203, 1006 BE Amsterdam, the Netherlands; 10 Department of Genetics, University Medical Center, University of Groningen, PO Box 30001, 9700 RB Groningen, the Netherlands

**Keywords:** Ovarian carcinoma, Genetic counseling, Psychological wellbeing, Genetic testing

## Abstract

The new Dutch guidelines on hereditary and familial ovarian carcinoma recommend genetic testing of all patients with epithelial ovarian cancer (EOC). With this study, we aimed to obtain insight into (1) the acceptance and timing of the offer of genetic counseling in women with EOC, (2) reasons for accepting or declining genetic counseling, and (3) psychological differences between women who did and did not have genetic counseling. A multicenter questionnaire survey was performed in patients with EOC in four Dutch oncology centers. The questionnaire addressed whether, how, and when genetic counseling was offered, women’s arguments to accept or decline genetic counseling, and included the Cancer Worry Scale (CWS) and the Hospital Anxiety and Depression Scale (HADS). A total of 67 women completed the questionnaire, of which 43 had genetic counseling. Despite a wide variability in the timing of the offer of genetic counseling, 89% of the women were satisfied with the timing. No significant differences were found between the CWS and HADS scores for the timing of the offer of genetic counseling and whether or not women had genetic counseling. Taking the small sample size into account, the results tentatively suggest that genetic counseling may have limited impact on the psychosocial wellbeing of women with EOC. Therefore, we assume that implementation of the new guidelines offering genetic counseling to all patients with EOC will not cause considerable additional burden to these patients.

## Introduction

Epithelial ovarian, peritoneal, and fallopian tube carcinoma (EOC) is the seventh most common cancer affecting women worldwide. In 2012, approximately 239,000 women worldwide were diagnosed with EOC and 151,000 women died of EOC that same year (Ferlay et al. 2012). In the Netherlands, 1200 to 1300 women are diagnosed with EOC every year (Dutch Cancer Registry 2015). EOC is often diagnosed at an advanced stage and is then associated with a poor prognosis. The 5-year survival of high-grade serous EOC is 20–60% (IKNL 2012). Around 10–20% of EOC is hereditary, of which the *BRCA1* and *BRCA2* genes account for the majority (Alsop et al. 2012; Walsh et al. 2011; Zhang et al. 2011). A minority of hereditary EOC is caused by germline mutations in other genes, e.g., *RAD51D*, *RAD51C*, *BRIP1*, and the mismatch repair genes (Lynch syndrome) (Loveday et al. 2011; Malander et al. 2006; Meindl et al. 2010; Rafnar et al. 2011). Identification of a germline mutation facilitates the identification of healthy family members with a mutation in EOC susceptibility genes. In these individuals, prevention of EOC and related types of cancer can be lifesaving. Furthermore, nowadays, identification of germline mutations may have implications for the treatment of women with EOC, since PARP inhibitors are most effective in women with recurrent EOC with a somatic or germline *BRCA1* or *BRCA2* mutation (Mirza et al. 2016). Although serum CA-125 measurements and transvaginal ultrasound have long been the recommended screening tools for women at increased risk for EOC, those screening methods have been proven ineffective (Hermsen et al. 2007; van der Velde et al. 2009). Nowadays, a bilateral risk-reducing salpingo-oophorectomy is recommended for female *BRCA* mutation carriers (Rebbeck et al. 2009). A majority of serous EOC appears to originate from dysplastic lesions in the distal fallopian tube; ongoing studies will demonstrate whether salpingectomy leads to delayed choice for oophorectomy (Erickson et al. 2013). In patients with EOC, a substantial part of the germline *BRCA1/2* mutation carriers are missed when applying criteria for genetic testing, such as age of onset, family history, and histology (Arts-de Jong et al. 2016). Therefore, the recently accepted Dutch guidelines on EOC recommend genetic counseling and the offer of a DNA test for *BRCA1* and *BRCA2* to all patients with EOC, irrespective of histology, family history, or age at diagnosis. Depending on personal and family history, complementary genetic tests (e.g. for Lynch syndrome) can be considered (IKNL 2015). In line with this, other international guidelines also advise to offer genetic counseling to all patients with EOC, e.g., in the USA (NCCN Guidelines version 1.^2018^: Breast and/or ovarian cancer genetic assessment). Concerns may rise about the burden and timing of offering genetic counseling to this group of women. In patients with EOC from different countries, acceptance rates of genetic counseling range from 60–99% (Alsop et al. 2012; Dekker et al. 2013; Majdak et al. 2005; Malander et al. 2006; Pal et al. 2005; Yazici et al. 2002). In the only available Dutch study, 25 of 35 patients with EOC accepted genetic testing. The lack of a correlation between the acceptance rate and time since diagnosis suggests that timing did not influence the uptake of genetic counseling (Dekker et al. 2013).

So far, little is known about the psychosocial impact of genetic counseling of women who have recently been diagnosed with EOC. One study investigating the psychosocial impact of genetic testing for *BRCA* mutations found no increase in general anxiety, depression, or psychological distress during genetic counseling or 1 year afterwards in patients with a personal or family history of breast cancer or EOC (Bish et al. 2002). Others reported that offering genetic counseling to breast cancer patients during treatment did not lead to short-term or long-term adverse effects (Baars et al. 2014; Ringwald et al. 2016; Schlich-Bakker et al. 2008). To our knowledge, only one study has focused on the timing of genetic counseling in patients with EOC so far (Bjornslett et al. 2015). This latter study used the Multidimensional Impact of Cancer Risk Assessment (MICRA) in a large group of patients with EOC that underwent genetic counseling. The MICRA measures the specific impact of result disclosure after genetic testing. The authors reported that cancer-related distress and a positive *BRCA* result had the strongest association with the MICRA scores. However, the time from diagnosis/disclosure to the survey was not related to the MICRA score.

The present questionnaire-base study assessed the burden and timing of offering genetic counseling to women in the Netherlands with EOC. The aim was to obtain insight into whether women with EOC are inclined to accept or decline genetic counseling, their reasons for doing so, their views on the timing of the offer in the course of their disease, by which professional genetic counseling should be offered and what possible adverse psychological effects might result from genetic counseling.

## Methods

### Participants

In this multicenter study, we used a questionnaire for eligible patients with EOC, 6–12 months after their initial diagnosis. Inclusion criteria were a recent diagnosis or recurrence of EOC. Excluded were patients with a known genetic predisposition for EOC and/or inadequate proficiency in Dutch language.

### Procedures

Between April 2014 and December 2015, consecutive patients with EOC from four Dutch oncology centers (University Medical Center Groningen; Amsterdam UMC, location AMC; the Netherlands Cancer Institute Amsterdam; Radboudumc Nijmegen) were informed about the study by their gynecologist and received an information sheet and informed consent form. On the informed consent form, women could indicate whether they consented to receive a questionnaire around 6 months after their initial diagnosis. They were asked to return the consent form to the investigators by mail (an envelope was provided). Due to practical constraints, we could not record how many information sheets and informed consents were handed out. Consenting women received a paper or digital questionnaire, depending on the woman’s preference. The local Medical Ethical Committee deemed that the Medical Research Involving Human Subjects Act was not applicable for this study. Therefore, a formal review by the Medical Ethics Committee was not required.

### Instrumentation

The questionnaire consisted of three parts (A, B, C). In part A, demographic and medical information (moment of diagnosis, treatment, personal history, and family history of cancer) were collected. Part B consisted of questions about whether, how, and when genetic counseling was offered and reasons to accept or decline genetic counseling. Genetic counseling was defined as any consultation at the department of clinical genetics, irrespective of whether a DNA test was performed. Reasons to accept or decline genetic counseling were inventoried by providing a checklist with response options (see Table 3 for items) supplemented with an “open text” box to allow for additional reasons. Patients were also asked for their opinion regarding the offer of genetic counseling (answered on a 5-point Likert scale). The questionnaire did not address the outcomes of genetic counseling, and we had no informed consent to collect these data. Part C consisted of the Dutch versions of the Cancer Worry Scale (CWS) and the Hospital Anxiety and Depression Scale (HADS). The CWS is a validated scale to assess cancer-specific distress, consisting of eight items answered on a 4-point Likert scale, resulting in scores ranging from 8 to 32 (Douma et al. 2010; Lerman et al. 1994; Lerman et al. 1991). The higher the score, the higher the level of distress. The HADS is a validated 14-item questionnaire investigating anxiety (HADS-A) and depression (HADS-D) (Spinhoven et al. 1997; Zigmond and Snaith 1983). Both subscales of the HADS consist of seven items answered on a 4-point Likert scale, resulting in scores from 0 to 21. Higher scores indicate a higher level of anxiety or depression. In the present study, Cronbach’s alphas for the CWS, HADS-A, and HADS-D were 0.92, 0.86, and 0.83, respectively, implying good internal consistency.

### Data analysis

For this study, only available data were analyzed and any missing data, due to some questions not being answered, were ignored. An independent samples *t* test was used to compare the age of women who did and who did not return their questionnaire. Descriptive statistics were used to explore the demographic and medical characteristics of the participants. Chi-squared tests were used to test for differences between women who had genetic counseling and those who did not, regarding age, education level, previous cancer, presence of a first-degree family member with cancer, the moment in treatment and timing of the offer of genetic counseling. To address psychological wellbeing and possible adverse effects of genetic counseling, independent *t* tests and one-way analyses of variance in case of > 2 groups were used to compare differences in the CWS scores between groups differing in the timing of the offer of genetic counseling and whether or not women had genetic counseling. Since the outcomes of the HADS-A and HADS-D, both in the entire group and in some of the subgroups, were not normally distributed (assessed visually and by the Shapiro-Wilk test), the Mann-Whitney *U* test and the Kruskal-Wallis test were used to compare the HADS scores. All data were analyzed using SPSS for Windows (version 19.0; SPSS; IBM Corp).

## Results

A total of 91 women provided informed consent. Of these women, 90 (99%) consented to receive a questionnaire and 67/90 (74%) returned their (partially) completed questionnaire.

### Part A: patient characteristics

General characteristics of the participating women are presented in Table 1. Mean age of the participants was 64 years (SD 11.7), which was not significantly different from the women who did not return their questionnaire (64 vs 67 years; *p* = 0.29). A medical history of cancer, besides EOC, was reported by 30% of the women (8 with breast cancer, 8 skin cancer, 1 pancreatic cancer, 1 cervical cancer, 1 stomach cancer, and 1 with kidney cancer).

**Table 1:** Comparison of EOC patients who did and did not have genetic counseling^a^

		Total	Genetic counseling	No genetic counseling	*P* value
Age	> 60	47	29	16	0.30
	≤ 60	20	14	4	
Education	Primary/middle	43	28	12	0.79
	High/university	22	14	7	
Previous cancer	No	46	27	15	0.21
	Yes	20	16	4	
First degree relative with cancer	No	22	16	5	0.34
	Yes	45	27	15	
Treatment finished	No	16	13	2	0.08
	Yes	51	30	18	
Genetic counseling discussed	Before start of treatment	21	17	4	0.47
	During treatment	22	14	7	
	After treatment	14	9	5	
Wonder about cancer being hereditary	Never	13	7	6	0.32
	Yes, sometimes	30	21	9	
	Yes, often	19	15	4	

^a^Subgroups do not all count up to 67 as the result of missing data

### Part B: experience with genetic counseling

Of the 67 participants, 43 received genetic counseling. There were no significant differences in age, educational level, previous cancer, presence of a first-degree family member with cancer, moment in treatment, moment of the offer of genetic counseling, and how often women wonder about their cancer being hereditary between women who received genetic counseling and women who did not (yet) receive genetic counseling (Table 1). Of the 20 women who did not have genetic counseling yet, 12 reported they were planning to do so within 6 months, 2 were planning to do so within a period longer than 6 months, 4 were not planning to undergo genetic counseling, and 2 had not yet decided.

**Fig. 1:**
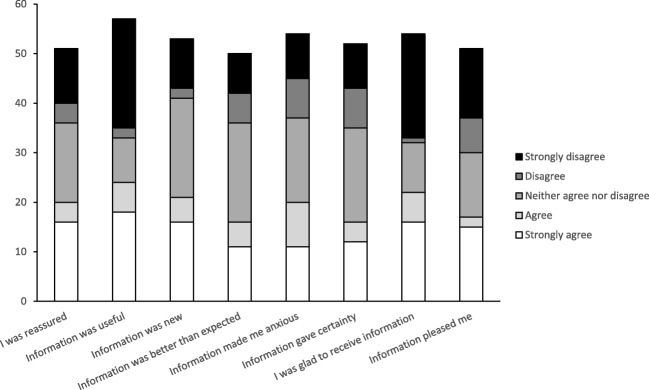
Patients’ reaction to the offer of genetic counseling

Regarding the offer of genetic counseling, for 29 women, this was offered by the gynecologist, for 12 by the clinical geneticist, and 8 women reported that both the gynecologist and clinical geneticist offered genetic counseling. Very few women were informed by their general practitioner (*n* = 2), radiotherapist (*n* = 1), surgeon (*n* = 5), nurse (*n* = 2), and/or family members (*n* = 6). Ten women reported that genetic counseling was not offered to them. Of those who received an offer, 98% appreciated the offer and felt that they had been informed by the appropriate person. Nevertheless, women felt very different about the offer of genetic counseling (Fig. 1). For example, 24 women felt the information they received about genetic counseling was not useful. Furthermore, 22 women would rather have not received the information about genetic counseling. All except two women were satisfied with the timing of the offer of genetic counseling. The two dissatisfied women preferred to postpone the referral until after treatment and one of these women preferred to wait until a few months later (Table 2). Participants were also asked about their considerations regarding whether to accept or decline genetic counseling, irrespective of their own final decision (Table 3).

**Table 2:** Women’s opinion about the timing of the offer for genetic counseling

Moment genetic counseling was offered	Good timing	Suboptimal timing	Do not know	Total
At diagnosis	3	0	1	4
Before start of treatment	17	0	0	17
During CT, before surgery	6	0	1	7
During CT, after surgery	12	1	1	14
After completion of treatment	13	1	0	14
Do not remember	0	0	1	1
Total	51	2	4	57

*CT* chemotherapy

**Table 3:** Reasons to accept or decline genetic counseling^b^

Arguments to accept genetic counseling	*n* (%)	Arguments to decline genetic counseling	*n* (%)
To discover cancer early in myself or family members For my children For my family It is important for future treatment To know the cause of my ovarian cancer To have role in cancer prevention myself Because my family will appreciate it More information gives me the feeling of having control To be certain It is doctor’s advice	52 (78) 47 (70) 32 (48) 31 (46) 30 (45) 20 (30) 19 (28) 18 (27) 14 (21) 9 (13)	Small chance of a hereditary cause It is not important for treatment There are many other things to care about Afraid for results I do not feel to think about my cancer risk I think my family gets worried It will make me anxious Insurance consequences for me and family I have no children I do not want to go to hospital more often It is too time consuming My family does not want me to do it	16 (24) 9 (13) 8 (12) 7 (10) 7(10) 6 (9) 6 (9) 5 (7) 4 (6) 3 (4) 2 (3) 1 (1)

*n* number of participants reporting this argument

^b^All reasons listed were choices presented in the questionnaire. Participants could select any that they felt applied. No participants added additional reasons in the open text box

### Part C: psychological distress

The mean score on the CWS was 17.0 (SD 5.5). The median score on the HADS-A was 6.0 (range 0–13) and on the HADS-D was 4.0 (range 0–15). Table 4 presents comparisons between the CWR and HADS scores for the timing of the offer of genetic counseling and whether or not women had genetic counseling. There were no significant differences in these items. Also, there were no significant differences in the CWS, HADS-A, and HADS-D scores depending on age, level of education, presence of a first-degree relative with cancer or the moment in treatment (data not shown). Interestingly, women who reported to have a history of another type of cancer had significantly lower scores for cancer worry, anxiety, and depression: *p* values were < 0.001, 0.001, and 0.011 for the CWS, HADS-A, and HADS-D, respectively.

**Table 4:** Cancer worry, anxiety, and depression according to timing of the offer for genetic counseling and whether or not women had genetic counseling

		CWS				HADS-anxiety				HADS-depression			
Variable		Frequency	Mean	SD	p value	Frequency	Median	Range	p value	Frequency	Median	Range	p value
Timing of offer of genetic counseling	Before start of treatment	20	17.8	5.2	0.32	20	6.5	0–13	0.24	21	5.0	0–12	0.77
	During treatment	22	18.2	5.7		20	6.0	0–12		21	5.0	0–10	
	After treatment	14	15.4	5.7		14	3.5	0–11		14	4.0	0–15	
Had genetic counseling	No	20	17.8	5.6	0.48	18	7.0	0–12	0.22	19	4.0	0–12	0.66
	Yes	41	16.7	5.6		42	5.0	0–13		43	3.0	0–15	

## Discussion

In this study, the participants generally appreciated the offer of genetic counseling and were content with the timing of the offer, and the uptake of genetic counseling 6–12 months after the diagnosis was high. No association was found between psychological distress and the moment of discussing genetic counseling. Furthermore, no difference was found in psychological distress between women who received genetic counseling and those who had not (yet) received genetic counseling. Therefore, there seem to be no psychological objections to comply with the new Dutch guidelines.

Most women reported that genetic counseling was offered by their gynecologist. This is as expected since, in the Netherlands, the gynecologist makes the diagnosis and coordinates treatment. However, 12 women reported that they received the offer for genetic counseling from their clinical geneticist. This is unexpected, since women have access to clinical genetic care only after referral by another clinician. It is unlikely that referral takes place without mentioning this to the patient. However, it is possible that the women had forgotten that they had received information about genetic counseling from the gynecologist or another referring clinician. This issue has been reported earlier (Vogel et al. 2018). Also, the referring clinician may not have spent sufficient time on the subject or the women may have forgotten the information due to the high impact of the diagnosis and/or treatment of EOC. Therefore, some women may have (erroneously) experienced being informed by the clinical geneticist only.

A relatively large proportion of women reported that the offer for genetic counseling was not useful to them and that they would rather have not received this information. This might be a worrisome finding and seems conflicting with the fact that 98% appreciated the offer for genetic counseling. Although we did not ask why women felt this way, they may have experienced insufficient quality of the information supply, and/or the content of the information they received. It may also have to do with the fact that genetic counseling could not take place at the moment of receiving the information. Further research is required to further understand these reactions.

At the moment of completing the questionnaire, 68% of the participants had received genetic counseling and most of the remaining women were considering genetic counseling in the near future. Only four women were not considering genetic counseling. This high uptake of genetic counseling is in line with other literature (Alsop et al. 2012; Dekker et al. 2013; Majdak et al. 2005; Malander et al. 2006; Pal et al. 2005; Yazici et al. 2002). That most women were planning to have genetic counseling in the near future might be reflected in the lack of a difference in the variables presented in Table 1. Previous studies reported that (as in our study), in patients with EOC and colorectal carcinoma, the most common reasons to accept genetic counseling are that patients want to obtain information on their own cancer risk and that of family members and to receive appropriate surveillance advice (Dekker et al. 2013; Esplen et al. 2007). In women with EOC, reasons to decline genetic counseling were the absence of relatives for whom genetic counseling would be relevant, being too ill to come to the hospital or being not willing to come to the hospital (Dekker et al. 2013; Pal et al. 2005). Emotional concerns, such as being afraid of the test result and worrying family members, did not emerge as relevant in these latter studies but were mentioned in focus groups by patients with breast cancer and EOC (Kne et al. 2017; Vogel et al. 2018). In the present study, the women also mentioned emotional reasons. It is important that clinicians are aware of these considerations and discuss them either before or after referral to a genetics department.

In the present study, for the four women not considering genetic counseling, their reasons for declining genetic counseling were making their family worried (*n* = 2), having other things to care about (*n* = 2), family does not want it (*n* = 1), having no children (*n* = 1), small chance of hereditary cause (*n* = 1), and not wanting to go to the hospital again (*n* = 1). Interestingly, they did not mention being afraid of the result or being anxious themselves, and they scored relatively low on cancer worry, anxiety, and depression (CWS mean 13.8; HADS-A median 2; HADS-D; median 2.5). These women might be less worried in general or less worried about their cancer being hereditary. Therefore, they might be less inclined to opt for genetic counseling. However, more research is needed to substantiate this assumption. Ideally, all patients declining genetic counseling should be empowered to make an informed decision. For example, patients who have no children might have other family members at risk for (ovarian) cancer or the patients mentioning a small chance of a hereditary cause might underestimate their risk. Indeed, it has been shown that cancer patients may have a lack of knowledge regarding genetics (Dekker et al. 2013; Geer et al. 2001; Vogel et al. 2018). Therefore, it is important that the referring physician thoroughly explores a woman’s reasons for declining genetic counseling. Interventions, such as patient information sheets and/or digital tools to support decision-making, might help tackle this issue (Grimmett et al. 2018).

Guidelines on genetic counseling for patients with EOC do not indicate the best moment to offer and plan genetic counseling (IKNL 2015; NCCN Guidelines version 1.^2018^: Breast and/or ovarian cancer genetic assessment). In the present study, most patients received the offer for genetic counseling shortly after the diagnosis of EOC and before the start of treatment. However, there was considerable variability in the timing. In a previous study on Dutch patients with breast cancer, no adverse psychological effects were reported after genetic counseling and testing that occurred shortly after diagnosis and before treatment (Wevers et al. 2011). Similarly, we found no significant difference in distress and anxiety scores for the different moments of discussing genetic counseling. When evaluating the timing of the offer of genetic counseling, almost all women were satisfied with when it occurred. Altogether, this suggests that there is no “best” moment to discuss genetic counseling or that caretakers tailor the timing of information provision to the needs of their individual patients. Moreover, after having received the offer, women choose the timing of the actual genetic counseling themselves.

Of note, in the present study, the overall median anxiety and depression scores in this study (6.0 and 4.0 respectively) are higher than reported for the general population and in cancer patients. For example, in a random sample of Dutch individuals aged 57–65 years, the mean HADS-A score was 3.9 and the mean HADS-D score was 3.7 (Spinhoven et al. 1997). A study among women with breast cancer or a gynecologic cancer reported a mean HADS-A score of 3.9 24 weeks after surgery (Stafford et al. 2013). In women with cervical carcinoma, the mean HADS-A score was 5.6 and 5.4 (early stage and locally advanced respectively) and the HADS-D score was 2.7 and 3.1 6 months after surgery (Ferrandina et al. 2012). In the present study, the mean CWS score (17.0) is also higher than previously reported in other types of cancer. In a study on breast cancer patients, the CWS score was 13.7 and 14.5 (rapid genetic testing and usual care respectively) 6 months after diagnosis. Although no CWS or HADS scores specific for EOC are available, they might be higher than in other types of cancer because of the generally worse prognosis of EOC. Higher scores might also reflect a response bias, with more distressed women being more inclined to participate in a study aimed at assessing their experiences. Strikingly, in our study, all distress scores were significantly lower in the group of women who reported a history of another type of cancer. To our knowledge, no previous studies have reported a similar finding. A possible explanation might be that women with a previous malignancy had acquired coping strategies which they effectively apply when a second malignancy is diagnosed.

With the extension of the indication for genetic testing in patients with EOC and the rapidly changing treatment of EOC, the timing and implementation of genetic counseling in these patients may change. Knowing the germline *BRCA* status before start of treatment may become increasingly important (George et al. 2016). In this light, our results suggest that genetic testing can be safely discussed shortly after diagnosis. Furthermore, options such as genetic testing by the gynecologist can also be explored.

The present study has several limitations. Based on the experience of the gynecologists, we assume that a relatively large number of patients did not return the informed consent form. However, because we were unable to record how many information sheets and informed consents were handed out and to which patients, we do not know whether there is a difference between participating and non-participating women. We estimate that about 500 women were treated for EOC in the participating hospitals during the study period, resulting in an estimated inclusion rate of 18% (90/500) of all eligible women. Some selection bias may have occurred since women with a positive feeling about genetic testing or those with a relatively good prognosis might have been more inclined to participate. On the other hand, the relatively high scores on the CWS and HADS might indicate a bias of more distressed women being inclined to participate. A bias might have led to an over- or underestimation of the uptake of genetic counseling and the effect of genetic counseling on cancer worry, anxiety, and depression. On the other hand, the bias might be small since the uptake of genetic counseling was comparable with earlier studies and there are no indications for a trend towards an effect of genetic counseling on cancer worry, anxiety, and depression. If less worried women are less inclined to opt for genetic counseling, as mentioned above, a bias towards more women interested in genetic counseling might be a contributing factor in the generally high scores on the CWS and HADS. Unfortunately, we did not record the outcomes of genetic testing, including whether women chose for a DNA test and the result of the test. These results could have influenced our findings. Other factors to consider when interpreting the results are the relatively small sample size and the fact that some of the questions addressed events in the past, possibly leading to a recall bias.

In conclusion, offering genetic counseling after the diagnosis of EOC appears to have limited impact on the psychosocial wellbeing of these patients. Therefore, we assume that implementation of the new Dutch guideline on hereditary EOC causes no unjustified additional burden to women with EOC. However, more systematic prospective studies are needed to confirm our results. Investigation of other hypotheses from this study may include the generally high scores on the CWS and HADS in patients with EOC and the lower scores in patients who declined genetic counseling.
